# Клинико-иммунологические предикторы течения эндокринной офтальмопатии после радиойодтерапии болезни Грейвса

**DOI:** 10.14341/probl13293

**Published:** 2023-11-10

**Authors:** М. С. Шеремета, Е. Г. Бессмертная, А. Р. Елфимова, Д. М. Бабаева, И. М. Беловалова, Н. Ю. Свириденко

**Affiliations:** Научный медицинский исследовательский центр эндокринологии; Научный медицинский исследовательский центр эндокринологии; Научный медицинский исследовательский центр эндокринологии; Научный медицинский исследовательский центр эндокринологии; Научный медицинский исследовательский центр эндокринологии; Научный медицинский исследовательский центр эндокринологии

**Keywords:** болезнь Грейвса, эндокринная офтальмопатия, радиойодтерапия, цитокины, антитела

## Abstract

**ОБОСНОВАНИЕ:**

ОБОСНОВАНИЕ. Данные о влиянии 131I на течение эндокринной офтальмопатии (ЭОП) противоречивы. Ряд исследований свидетельствует об ухудшении течения ЭОП на фоне проведения радиойодтерапии (РЙТ), в других исследованиях такой связи не установлено. Цитокины, регулирующие воспаление, потенциально могут быть биомаркерами для оценки активности ЭОП и прогноза течения ЭОП после РЙТ.

**ЦЕЛЬ:**

ЦЕЛЬ. Исследование динамики глазных симптомов и анализ иммунологических показателей: цитокина TGF-β1 и рецепторов цитокинов: sTNFα-R1, sTNFα-R2, sIL-2R, sIL-6R в разные сроки после РЙТ как возможных предикторов активации ЭОП.

**МАТЕРИАЛЫ И МЕТОДЫ:**

МАТЕРИАЛЫ И МЕТОДЫ. В исследование были включены 59 пациентов (118 орбит) с болезнью Грейвса (БГ) в состоянии эутиреоза и субклинического тиреотоксикоза и ЭОП в низко активной и неактивной фазе, направленных на проведение РЙТ. Определены концентрации цитокина TGF-β1, sTNFα-RI и sTNFα-R2, sIL-2R, sIL-6R, антител к рецептору ТТГ (рТТГ), свободных фракций тироксина (свТ4) и трийодтиронина (свТЗ), тиреотропного гормона (ТТГ) в сыворотке крови. Выполнено ультразвуковое исследование щитовидной железы (УЗИ ЩЖ), мультиспиральная компьютерная томография (МСКТ)/магнитно-резонансная томография (МРТ) орбит. Обследование проводилось через 3, 6, 12 мес после проведения РЙТ.

**РЕЗУЛЬТАТЫ:**

РЕЗУЛЬТАТЫ. Ухудшение течения ЭОП (всего на 1–2 балла по CAS) отмечено через 3 мес (32,5%) и в меньшем проценте через 6 и 12 мес (13,2 и 8,45% соответственно). Динамики не отмечено примерно у одинакового количества пациентов (40,5, 41,5, 45,8% соответственно). Улучшение течения ЭОП отмечено через 6 и 12 мес (45,3, 45,8% соответственно). Через 3 и 6 мес отмечено развитие гипотиреоза и достоверное повышение уровня антител к рТТГ. При анализе цитокинов и их рецепторов отмечено достоверное снижение уровня TGF-β1 через 3, 6 и 12 мес. Также отмечено достоверное снижение sTNF-R1 и sIL-2R через 3 и 6 мес. Уровень sTNFα-R2 достоверно снизился через 3 мес после РЙТ. Уровень sIL-6R существенно не изменился. Через 3 мес у пациентов с положительной динамикой ЭОП уровень TGF-β1 существенно не изменился по сравнению с уровнем до РЙТ, у пациентов с ухудшением течения ЭОП или без динамики уровень TGF-β1 достоверно снизился от исходного. Через 6 мес имелась та же тенденция, не достигающая статистической значимости. Уровень IgG4 и отношение IgG4/IgG, %, повысились к 6 и 12 мес, что коррелировало с количеством пациентов с диплопией.

**ЗАКЛЮЧЕНИЕ:**

ЗАКЛЮЧЕНИЕ. Главным лимитирующим фактором проведения РЙТ является активность аутоиммунного процесса в орбитах. Так как на проведение РЙТ направлялись пациенты в неактивной фазе ЭОП (CAS 0–2) или в состоянии низкой активности (CAS 3–4), выраженной активации ЭОП после РЙТ не произошло. Отмечено незначительное ухудшение течения ЭОП всего на 1–2 балла по CAS через 3 мес (32,5%) и в меньшем проценте — через 6 мес (13,2%). В ходе проведенного исследования установлено, что основными предикторами ухудшения течения ЭОП после РЙТ являются некомпенсированный гипотиреоз, высокий уровень антител к рТТГ и снижение уровня цитокина TGF-β1.

## Abstract (en)

**BACKGROUND:**

BACKGROUND: Data on the effect of 131I on the course of Graves’ orbitopathy (GO) are contradictory. A number of studies indicate a deterioration in the course of GO against the background of RAIT, in other studies such a connection has not been established. Cytokines that regulate inflammation could potentially be biomarkers for assessing GO activity and predicting the course of GO after RAIT.

**AIM:**

AIM: The purpose of this study was to evaluate the dynamics of eye symptoms and analyze immunological parameters: cytokine TGF-β1 and cytokine receptors: sTNFα-R1, sTNFα-R2, sIL-2R, sIL-6R over time after RAIT, as possible predictors of GO activation.

**MATERIALS AND METHODS:**

MATERIALS AND METHODS: The study included 59 patients (118 orbits) with GD in the state of euthyroidism and subclinical hyperthyroidism and low active and inactive GO, aimed at conducting RAIT. Concentrations of cytokine TGF-β1, sTNFα-RI and sTNFα-R2, sIL-2R, sIL-6R, TSH receptor antibodies (rTSH-Ab), free thyroxine (FT4) and free triiodothyronine (FT3), ­thyroid-stimulating hormone (TSH) in the blood serum were determined. Ultrasound examination of the thyroid gland, multispiral computed tomography (MSCT)/magnetic resonance imaging (MRI) of the orbits was performed. The examination was carried out 3, 6, 12 months after the RAIT.

**RESULTS:**

RESULTS: The deterioration of the course of the GO (1–2 points according to CAS) was noted after 3 months. (32.5%) and to a lesser degree after 6 and 12 months (13.2% and 8.45%, respectively). Dynamics were not noted, approximately, in the same number of patients (40.5%, 41.5%, 45.8%, respectively). An improvement in the course of the GO was noted after 6 and 12 months (45.3, 45.8, respectively). After 3 and 6 months, the achievement of hypothyroidism and a significant increase in the level of rTSH-Ab were noted. In the analysis of cytokines and their receptors a significant decrease in the level of TGF-β1 was noted after 3, 6 and 12 months. There was also a significant decrease in sTNF-R1 and sIL-2R at 3 and 6 months. The level of sTNFα-R2 significantly decreased 3 months after RAIT. The level of sIL-6R has not changed significantly. After 3 months in patients with positive dynamics of image intensification, the level of TGF-β1 did not significantly change compared with the level before RAIT, in patients with worsening of the course of GO or without dynamics, the level of TGF-β1 significantly decreased. After 6 months, there was the same trend, not reaching statistical significance. The IgG4 level and the IgG4/IgG ratio increased to 6 and 12 months, which corresponded to an increase in diplopia index.

**CONCLUSION:**

CONCLUSION: The main limiting factor in the conduct of RAIT is the activity of the autoimmune process in the orbits. Since patients with inactive (CAS 0–2) or low activity (CAS 3–4) GO were referred for RAIT, there was no pronounced activation of GO after RAIT. There was a slight deterioration in the course of GO by only 1–2 points according to CAS after 3 months. (32.5%) and to a lesser degree after 6 months (13.2%). In the study, it was found that the main predictors of the deterioration of the course of GO after RAIT are uncompensated hypothyroidism, a high level of rTSH-Ab and a decrease in the level of cytokine TGF-β1.

## АКТУАЛЬНОСТЬ

Радиойодтерапия (РЙТ) — эффективный, безопасный и экономически выгодный метод лечения пациентов с болезнью Грейвса (БГ) [1]. Целями РЙТ являются ликвидация тиреотоксикоза путем разрушения гиперфункционирующей ткани щитовидной железы (ЩЖ) и достижение стойкого гипотиреоидного состояния. Под действием ионизирующего излучения происходит нарушение структуры ЩЖ и разрушение тиреоцитов. Гипотиреоз может развиваться в период от 4 нед (у 40% пациентов развитие гипотиреоза отмечается к 8-й неделе и у более чем 80% — к 16-й неделе) [2]. Связь между РЙТ БГ и развитием эндокринной офтальмопатии (ЭОП) de novo или обострением уже имеющейся ЭОП широко обсуждается. В последние годы было проведено значительное количество исследований, направленных на изучение влияния РЙТ на течение ЭОП. В ряде исследований к факторам риска ухудшения течения ЭОП относят исходный некомпенсированный тиреотоксикоз до проведения РЙТ, высокий уровень антител к рТТГ, объем ЩЖ, курение, персистенцию тиреотоксикоза и некомпенсированный гипотиреоз в постлучевом периоде [3–16]. Последнее десятилетие характеризуется ростом интереса к изучению патогенетической роли цитокинов в формировании воспалительных заболеваний и определению перспектив иммуносупрессивной терапии. Цитокины, инфильтрирующие ретроорбитальные ткани, могут играть ключевую роль в развитии аутоиммунных реакций в орбите. Любой дисбаланс между выработкой про- и противовоспалительных цитокинов может вызвать аутоиммунный ответ с изменением сывороточных и локальных концентраций цитокинов, а также уровней их активности.

В предыдущем исследовании мы выявили высокие уровни солюбилизированных рецепторов цитокинов: sTNFα-R2, sTNFα-R2, sIL-2R и цитокина TGF-β1 у пациентов с длительно существующей нелеченой ЭОП и БГ в состоянии эутиреоза [17]. Уровень TGF-β1 был значимо выше у пациентов с ЭОП по сравнению со здоровыми лицами и повышался с увеличением длительности ЭОП. В последующем исследовании мы выявили более низкие показатели цитокина TGF-β1 исходно и в процессе лечения глюкокортикоидами у пациентов, резистентных к лечению, по сравнению с пациентами с положительной динамикой, что позволяет использовать TGF-β1 в качестве биомаркера активности процесса [18].

Целью настоящей работы явилось исследование динамики TGF-β1 и растворимых рецепторов цитокинов: sTNFα-R1, sTNFα-R2, sIL-2R, sIL-6R до и после проведения РЙТ как возможных предикторов неблагоприятного течения ЭОП после РЙТ.

## МЕТОДЫ

## Дизайн исследования

Проведено обсервационное одноцентровое проспективное контролируемое исследование.

## Критерии соответствия

В исследование были включены пациенты с БГ и ЭОП, верифицированными по международным стандартам диагностики [19]. Объектом исследования являлся пациент и его глаза. Учитывая то, что у одного и того же пациента глаза имеют разную степень выраженности клинических симптомов ЭОП, обработка результатов исследования проводилась отдельно для каждого глаза (шкала клинической активности — CAS) и каждой орбиты — МСКТ/МРТ. Тяжесть и активность ЭОП оценивались по наиболее пораженному глазу. Критериями исключения были сопутствующие аутоиммунные, хронические инфекционные заболевания, перенесенные вирусные заболевания в течение последних 3 мес, беременность и кормление грудью.

## Условия проведения

Исследование проводилось в ГНЦ РФ ФГБУ «НМИЦ эндокринологии» Минздрава РФ в отделе терапевтической эндокринологии и отделении радионуклидной терапии (РНТ).

## Продолжительность исследования

Включение пациентов в исследование проведено в 2019–2021 гг.

## Описание медицинского вмешательства

Всем пациентам проводились общеклиническое обследование со сбором анамнеза, физикальное обследование, определение уровней ТТГ, свТ4, свТЗ, антител к рТТГ, УЗИ ЩЖ с применением цветовой допплерографии. Всем пациентам было выполнено стандартное офтальмологическое исследование на базе отделения диабетической ретинопатии и офтальмохирургии ГНЦ РФ ФГБУ «НМИЦ эндокринологии» Минздрава России (ЭНЦ) (зав. отд. д.м.н. Липатов Д.В.): визометрия, определение уровня внутриглазного давления, биомикроскопия переднего отдела глаза, экзоофтальмометрия, компьютерная периметрия, биомикроскопия хрусталика и стекловидного тела с помощью щелевой лампы, обратная и прямая офтальмоскопия; визуализация орбит — МСКТ/МРТ. Диагноз ЭОП верифицировали соответственно рекомендациям EUGOGO [20]. Тяжесть ЭОП оценивалась по классификации NOSPECS [21]. Активность ЭОП оценивалась по шкале клинической активности CAS [21].

## Основной исход исследования

Проводились оценка клинических симптомов, определение антител к рТТГ, цитокинов и их растворимых рецепторов, показателей функциональной активности ЩЖ до и в динамике после РЙТ.

## Анализ в подгруппах

Для проведения исследования были сформированы группы до лечения и в разные сроки после проведения РЙТ (3, 6, 12 мес).

## Методы регистрации исходов

Содержание растворимых рецепторов sIL-6R, TGF-β1, IgG4 в образцах сыворотки определяли с помощью коммерческих наборов фирмы BenderMedSystems GmbH (Австрия), sTNFα-RI и TNFα-R2 — наборами R&Dsystems (США-Канада), IgG — набором Seramun DiagnosticGmbH (Германия), IL-2R — набором RayBiotech (США). Все вышеуказанные исследования выполняли методом иммуноферментного анализа, измерение оптической плотности проводили на счетчике 1420 Multilabel Counter VICTOR2 (Perkin Elmer). Уровни ТТГ, свТ3, свТ4 определяли методом усиленной хемилюминесценции на автоматическом анализаторе Architect (Аbbott Diagnostics, США). Референсные значения для базального ТТГ 0,25–3,5 мМЕ/л, свТ4 — 9,0–20,0 пмоль/л, свТ3 — 2,5–5,5 пмоль/л. Антитела к рТТГ (референсные значения 0,00–1,75 МЕ/л) определяли методом электрохемилюминесцентного анализа на иммунохимическом автоматическом анализаторе Cobas 6000 (Roche Diagnostics, Германия). Забор крови проводился утром натощак, после сна в условиях стационара. Лабораторные исследования проводили на базе клинико-диагностической лаборатории ГНЦ РФ ФГБУ «НМИЦ эндокринологии» Минздрава России (зав. лаб. к.м.н. Никанкина Л.В.). УЗИ ЩЖ выполнено на ультразвуковом сканере Toshiba Aplio 790 датчиком переменной частоты 7,5–10 МГц с применением цветовой допплерографии на базе отделения ультразвуковой диагностики ГНЦ РФ ФГБУ «НМИЦ эндокринологии» Минздрава России (зав. к.м.н. Солдатова Т.В.). МРТ, МСКТ орбит проводились в отделе лучевой диагностики НМИЦ (зав. к.м.н. Тарбаева Н.В.) на 320-срезовом аппарате Aquilion One (Toshiba, Япония) со сканированием в автоматическом режиме. Принимая во внимание возможность разной выраженности патологического процесса в обеих орбитах, каждую орбиту оценивали отдельно в 3 проекциях: аксиальной, корональной, сагиттальной. Определение минимальных и максимальных значений плотности экстраокулярных мышц и ретробульбарной клетчатки проводили в корональной и аксиальной проекциях, отступив 1–2 мм от контуров мышцы [15].

## Этическая экспертиза

Работа одобрена локальным Этическим комитетом при ГНЦ РФ ФГБУ «НМИЦ эндокринологии» Минздрава России. Протокол заседания локального этического комитета № 17 от 27.09.2018 г.

## Статистический анализ

Статистическая обработка данных выполнена на персональном компьютере с использованием пакета программ статистического анализа данных Statistica 6.13 (StatSoft Inc., США) и приложения Microsoft Exсel for Windows. Для количественных признаков рассчитывались: средние, минимальные и максимальные значения, стандартные отклонения (полученные результаты представлены в виде М±SD, где М — среднее арифметическое значение, SD — стандартное отклонение), либо медиана и квартили — Ме (10; 75; ). Анализ межгрупповых различий при нормальном распределении признака проводился с использованием t-критерия Стьюдента. Для сравнения независимых выборок при распределении признака, отличавшегося от нормального, использовался критерий Манна–Уитни–Уилкоксона. Критический уровень значимости при проверке статистических гипотез принимался равным 0,05 (p<0,05).

## РЕЗУЛЬТАТЫ

## Участники исследования

В исследование были включены 59 пациентов (118 орбит), в неактивной и низко активной фазе ЭОП и БГ, верифицированных по международным стандартам диагностики. Средний возраст составил 52,4±13,4 года в диапазоне от 21 до 70 лет, 59 женщин, 7 мужчин. До поступления в отделение РНТ пациенты имели показатели эутиреоза или субклинического тиреотоксикоза. Длительность лечения тиреотоксикоза до проведения РЙТ составила 30,5 [ 24; 96] мес (от 6 до 480 мес). Неактивную фазу ЭОП имели 76,6%, низкоактивную фазу имели 25,4%. Пациенты в активной фазе на РЙТ не направлялись. Легкую степень ЭОП имели 12,4%, среднюю 50,8%, тяжелую 6,8%. На снижение зрения жаловались 45,8%. Диплопия отмечалась у 30,5% и косоглазие у 11,9%. Клиническая и офтальмологическая характеристики пациентов с ЭОП и БГ до и после РЙТ представлены в таблице 1.

**Table:** Таблица 1. Клиническая и офтальмологическая характеристики пациентов с эндокринной офтальмопатией и болезнью Грейвса до и через 3, 6, 12 месяцев после проведения радиойодтерапии. Сравнение проводилось с исходными данными (0 точка) пациентов, продолжающих наблюдение Примечание: в скобках (*) 0 мес — сравнение с исходными данными.

Время, мес.	0	3	6	12
Количество пациентов/глаз	59/118	38/76	52/104	24/48
CAS (M±SD)	1,86±0,97	2,6±1,15р=0,426 (0 мес)*	1,38±0,94р=0,001 (0 мес)*	1,13±1,08р=0,033 (0 мес)*
CAS %0–2/3–4/5–7	74,6/25,4/0,0	68,4/31,6/0,0	84,3/15,7/0,0	91,6/8,42/0,0
Динамика глазных симптомов:ухудшениебез динамикиулучшение		32,5%40,5%27,0%	13,2%41,5%45,3%	8,4%45,8%45,8%
Тяжесть ЭОП Л/С/Т, %	42,4/50,8/6,8	32,3/61,8/5,9	40,0/54,0/6,0	36,4/63,6/0,0
Глазные симптомы
Боли при движении глаз/спонтанные боли	8,5%/10,1%	15,8%/13,2%	5,7%/5,7%	4,3%/0%
Краснота век	5,0%	7,9%	3,8%	6.,7%
Краснотаконъюнктивы	84,7%	78,9%	90,3%	73,9%
Отек век	45,8%	50,0%	26,9%	26,1%
Хемоз	28,8%	31,6%	17,3%	0,0%
Лагофтальм	18,6%	18,4%	13,5%	13,0%
Диплопия/косоглазие	30,5%/11,9%	34,2%/7,9%	28,8%/9,6%	34,8%/8,7
Снижение зрения	45,8%	44,7%	31,3%	25,4%
Экзофтальмометрия (M±SD)	19,65±2,77	19,52±3,25р=0,717 (0 мес)*	19,59±2,93р=0,454 (0 мес)*	18,72±3,39р=0,310 (0 мес)*
Щитовидная железа
Антитела к рТТГ, МЕ/л (M±SD)	13,87±11,3	21,41±12,8р=0,0003 (0 мес)*	17,90±14,2p=0,025 (0 мес)*	15,06±13,5p=0,736 (0 мес)*
ТТГ, мМЕ/л (M±SD)	1,91±4,17	16,68±25,27p=0,0002 (0 мес)*	5,3±9,02p=0,0004 (0 мес)*	2,38±2,58p=0,244 (0 мес)*
свТ4, пмоль/л (M±SD)	18,25±9,22	11,85±5,45p=0,0001p<0,001 (0 мес)*	13,40±3,08p=0,0008p<0,001 (0 мес)*	13,42±3,18p=0,020p<0,05 (0 мес)*
свТ3, пмоль/л (M±SD)	8,13±8,45	3,40±1,56p=0,0000 (0 мес)*	3,74±2,86p=0,0000 (0 мес)*	3,44±0,92p=0,0003 (0 мес)*
IgG, мкг/мл (M±SD)	9404,3±1744,1	9680,5±2029,5p=0,055 (0 мес)*	9195,2±2098,5p=0,780 (0 мес)*	9276,9±1855,3p=0,480 (0 мес)*
IgG4, мкг/млвыше нормы >1350 мкг/мл в %	35,1%	34,5%	33,3%	25%
IgG4, мкг/мл	1052,9±720,0	1186,3±846,1p=0,019 (0 мес)*	1087,0±836,2p=0,316 (0 мес)*	1219,4±972,8p=0,017 (0 мес)*
ОтношениеIgG4/IgG, %	65,2% >5% (от 5,1 до 28,5%)	78,9% >5% (от 5,1 до 31,8%)	68,2% >5% (от 5,5 до 35,2%)	85,7% >5% (от 5,3 до 40,9%)
Цитокины
TGF-β1, пг/млМедиана [ 25; 75]	28465,323738,6; 35102,1	21254,615878,3; 29838,1p=0,001 (0 мес)*	25336,420100,1; 30854,8p=0,044 (0 мес)*	19711,814560,7; 25600,0p=0,011 (0 мес)*
sTNFа-R1, пг/млМедиана [ 25; 75]	1210,51014,4; 1578,2	1063,1906,7; 1298,7p=0,0006 (0 мес)*	1157,1993,5; 1291,4p=0,0005 (0 мес)*	1096,0896,9; 1321,4p=0,679 (0 мес)*
sTNFα-R2, пг/млМедиана [ 25; 75]	2614,11874,2; 3109,1	2416,02037,5; 3375,1p=0,043 (0 мес)*	2304,71985,2; 3397,0p=0,139 (0 мес)*	1972,21666,5; 2944,1p=0,679 (0 мес)*
sIL-2R, пг/млМедиана [ 25; 75](M±SD)	104,752,4; 409,9	51,635,0; 54,8p=0,046 (0 мес)*	40,932,9; 144,5p=0,019 (0 мес)*	57,536,0; 106,5р=0,144 (0 мес)*
sIL-6R, пг/млМедиана [ 25; 75](M±SD)	176,0140,2; 202,2	158,6122,2; 183,0p=0,140 (0 мес)*	170,2141,5; 182,5p=0,125 (0 мес)*	173,2124,4; 224,7р=0,753 (0 мес)*

## ОСНОВНЫЕ РЕЗУЛЬТАТЫ ИССЛЕДОВАНИЯ

Главным лимитирующим фактором проведения РЙТ является активность аутоиммунного процесса в орбитах. На проведение РЙТ направлялись пациенты в неактивной фазе ЭОП (CAS 0–2) или в состоянии низкой активности (CAS 3–4). Пациентов в активной фазе ЭОП (CAS 5–7) лечили только консервативно. Средний показатель CAS (M±SD) составил 1,86±0,97 балла. Средний показатель ТТГ до РЙТ составил 1,91±4,17 балла.

Дозиметрическое обоснование (планирование) проводили по назначению врача-радиолога на системе ОФЭКТ Discovery с введением трейсерной активности радиофармацевтического лекарственного препарата (РФЛП) 131-йода, активностью от 5 до 10 МБк. Сцинтиграфия проводилась на 2 ч в режиме «Все тело» и на 24 ч «Статика». В рамках процедуры определяли индекс тиреоидного накопления 131-йода на 24 ч после введения трейсерной активности [%], уточняли объем долей ЩЖ по сцинтиграфическим признакам и по формуле 0,163 × (0,785×ширина правой доли (см) × длина правой доли (см))^(3/2). Терапевтическую активность рассчитывали для достижения целевой поглощенной дозы в ткани ЩЖ, равной 2 Гр/ч на 24 ч после введения.

Исследования проведены через 3, 6, 12 мес после РЙТ. Сравнительный анализ проводился попарно для пациентов, продолжающих наблюдение через 3, 6 и 12 мес (табл. 1). Эффективность лечения оценивалась по клиническим данным с учетом CAS и NOSPECS.

Ухудшение течения ЭОП (всего на 1–2 балла по CAS) отмечено через 3 мес (32,5%) и в меньшем проценте через 6 мес (13,2%). Динамики не отмечено примерно у одинакового количества пациентов (40,5, 41,5, 45,8% соответственно). Улучшение течения ЭОП отмечено через 6 и 12 мес (у 45,3 и 45,8% соответственно). Из глазных симптомов до РЙТ у пациентов преобладали краснота коньюнктивы у 84,7%, отек век у 45,8%, хемоз у 28,8%, снижение зрения у 45,8%. Через 3 мес усилились боли в глазах у 15,8%, хемоз у 31,6%, отек век у 50,0%, диплопия у 34,7%. Через 6 и 12 мес сохранялись в основном краснота конъюнктивы у 90,3 и 73,9% соответственно, отек век у 26,9–23,1%, диплопия у 28,8–34,8% пациентов соответственно. Показатели экзофтальмометрии практически не менялись. Через 3 мес достоверно увеличился уровень антител к рТТГ (p<0,001), через 6 мес уровень антител снизился, но оставался выше исходного (p<0,05). Через 12 мес уровень антител к рТТГ снизился к исходному. Через 3 и 6 мес отмечено развитие гипотиреоза. К 12 мес гипотиреоз был медикаментозно компенсирован.

При анализе цитокинов и их рецепторов отмечено достоверное снижение уровня TGF-β1 через 3, 6 и 12 мес (p<0,05, p<0,05, p<0,05 соответственно). Через 12 мес уровень TGF-β1 приближался к показателю здоровых лиц — 14320,7 [17]. Также отмечено достоверное снижение sTNF-R1 через 3 и 6 мес (p=0,0006, р=0,0005 соответственно) и снижение уровня sIL-2R через 3 и 6 мес (p<0,05, p<0,05 соответственно). Уровень sTNFα-R2 достоверно снизился через 3 мес после РЙТ (p<0,05). Уровень sIL-6R существенно не изменился. Уровень IgG4 и отношение IgG4/IgG, %, повысились к 6 и 12 мес, что соответствовало повышению показателя диплопии.

Мы проанализировали уровень цитокинов через 3 мес после РЙТ у пациентов с положительной динамикой глазных симптомов (10 пациентов; 24,6%), без динамики (14 пациентов; 36,8%) и у пациентов с ухудшением ЭОП (14 пациентов; 36,8%) (табл. 2).

**Table:** Таблица 2. Динамика цитокина TGFβ1, рецепторов цитокинов sTNF-R1, sTNFα-R2, sIL-2R, sIL-6R, антител к рТТГ через 3 месяца после радиойодтерапии в зависимости от течения ЭОП Примечание: в скобках (*) 0 мес+ — сравнение с исходными данными пациентов через 3 мес.

	Ухудшение14 пациентов	Без динамики14 пациентов	Положительная динамика10 пациентов
	0 мес	3 мес	0 мес	3 мес	0 мес	3 мес
CAS (M±SD)	1,86±0,87	3,46±0,69р=0,005(0 мес)*	1,33±0,70	1,33±0,70р=0(0 мес)*	2,66±0,81	1,50±0,54р=0,027(0 мес)*
Объем ЩЖ(M±SD)	28,9±15,5	13,89±0,89р=0,002(0 мес)*	33,2±24,2	16,7±10,2р=0,002(0 мес)*	25,1±19,6	11,0±5,9р=0,003(0 мес)*
ТТГ(M±SD)	1,36±2,23	29,26±38,99р=0,004(0 мес)*	0,68±1,52	12,2±14,5р=0,004(0 мес)*	3,90±6,8	9,42±5,5р=0,075(0 мес)*
Антитела к рТТГ(M±SD)	17,4±12,9	24,09±12,4р=0,049(0 мес)*	12,8±10,6	23,9±16,0р=0,017(0 мес)*	11,8±9,9	17,6±11,4р=0,091(0 мес)*
TGF-β1, пг/млМедиана [ 25; 75]	31556,025417,4; 35102,2	22666,919206,2; 30584,4р=0,008(0 мес)*	23600,017152,8; 26502,2	17700,015900,0; 19534,1р=0,028(0 мес)*	20088,512200,0; 32451,0	18575,511600,0; 25058,8р=0,916(0 мес)*
sTNFа-R1, пг/млМедиана [ 25; 75]	1223,191137,9; 2146,9	1157,71057,0; 1539,0p=0,027(0 мес)*	1157,01014,4; 1578,0	1123,0894,5; 1303,6р=0,524(0 мес)*	1124,5943,3; 1235,3	928,5726,0; 1141,0p=0,012(0 мес)*
sTNFα-R2, пг/млМедиана [ 25; 75]	2784,12162,2; 4627,4	2188,72062,7; 3378,0p=0,041,(0 мес)*	2487,81944,0; 3936,1	2827,02360,6; 4156,0p=0,878(0 мес)*	2535,91866,0; 3552,0	2179,51741,0; 3300,0p=0,138(0 мес)*
sIL-2R, пг/млМедиана [ 25; 75]	126,984,9:549,2	60,047,3; 143,5p=0,062, (0 мес+)*	99,2252,5:409,4	42,8235,0; 84,9p=0,500 (0 мес+)*	40,028,4:51,6	23,919,6; 28,4p=0,479 (0 мес)*
sIL-6R, пг/млМедиана [ 25; 75]	141,5128,5; 166,0	129,047,3; 143,5p=0,062, (0 мес)*	187,3117,7; 187,7	177,3163,7; 183,0p=0,500 (0 мес)*	157,0129,8; 184,3	148,3112,8; 183,8p=0,479 (0 мес)*

Через 3 мес у пациентов с положительной динамикой ЭОП уровень антител к рТТГ повысился на уровне статистических тенденций по сравнению с уровнем до РЙТ, у пациентов с ухудшением или без динамики уровень антител к рТТГ достоверно повысился. Уровень TGF-β1 имел противоположную динамику. У пациентов с положительной динамикой ЭОП уровень TGF-β1 существенно не изменился по сравнению с уровнем до РЙТ, у пациентов с ухудшением или без динамики уровень TGF-β1 достоверно снизился от исходного. Уровень sTNFа-R1 достоверно снизился у пациентов с ухудшением и улучшением, уровень sTNFα-R2 достоверно снизился у пациентов с ухудшением. Уровни sIL-2R и sIL-6R достоверно не изменились. Уровень ТТГ у пациентов с положительной динамикой ЭОП повысился, не достигая уровня статистической достоверности. У пациентов с ухудшением или без динамики значительно повысился уровень ТТГ. Объем ЩЖ во всех 3 группах достоверно снизился.

Через 6 мес после РЙТ самую большую группу составили пациенты с отсутствием динамики — 23 пациента (50,0%), с улучшением — 12 пациентов (26,1%), с ухудшением — 11 пациентов (23,9%). Объем ЩЖ продолжал уменьшаться во всех трех подгруппах, уровни антител к рТТГ оставались высокими у пациентов с ухудшением, повышались на уровне статистической тенденции у пациентов с положительной динамикой, не изменились у пациентов без динамики. Уровень ТТГ оставался высоким у пациентов с ухудшением, нормализовался у пациентов без динамики и снизился у пациентов с положительной динамикой до субклинических показателей. У пациентов с положительной динамикой ЭОП уровень TGF-β1 существенно не изменился по сравнению с уровнем до РЙТ, у пациентов с ухудшением или без динамики уровень TGF-β1 имел тенденцию к снижению. В группе с улучшением отмечено достоверное снижение уровня sTNFα-R1. Уровень sTNFα-R2 достоверно снизился в группе с ухудшением. Уровни sIL-2R и sIL-6R достоверно снизились у пациентов с ухудшением (табл. 3).

**Table:** Таблица 3. Динамика цитокина TGF-β1, рецепторов цитокинов sTNF-R1, sTNFα-R2, sIL-2R, sIL-6R, антител к рТТГ через 6 мес после радиойодтерапии в зависимости от течения ЭОП Примечание: в скобках (*) 0 мес+ — сравнение с исходными данными пациентов через 6 мес.

	Ухудшение12 пациентов	Без динамики23 пациента	Положительная динамика11 пациентов
	0 мес	6 мес	0 мес	6 мес	0 мес	6 мес
CAS (M±SD)	2,0±0,82	3,0±0,81р=0,017(0 мес)*	1,31±0,77	1,31±0,77р=0(0 мес)*	2,57±0,93	1,04±0,58р=0,00006(0 мес)*
Объем ЩЖ(M±SD)	28,3±11,1	7,62±4,19р=0,017(0 мес)*	28,9±23,7	9,1±8,7р=0,00002(0 мес)*	23,25±10,23	7,35±6,41р=0,0006(0 мес)*
ТТГ(M±SD)	2,25±5,1	11,51±15,35р=0,013(0 мес)*	0,75±1,32	2,5±3,9р=0,008(0 мес)*	2,03±4,8	6,39±9,57р=0,010(0 мес)*
Антитела к рТТГ(M±SD)	23,9±17,8	26,14±18,98р=0,017(0 мес)*	14,9±10,3	15,0±10,9р=0,493(0 мес)*	13,1±11,6	18,59±15,9р=0,056(0 мес)*
TGF-β1, пг/млМедиана [ 25; 75]	25417,024738,6; 27381,3	24159,922411,3; 26363,2р=0,500(0 мес)*	30839,917668,5; 35102,1	27278,417900,0; 33666,7р=0,751(0 мес)*	24888,9817600,0; 32451,0	24833,514800,0; 28075,4р=0,342(0 мес)*
sTNFа-R1, пг/млМедиана [ 25; 75]	1795,71247,0; 2564,2	1189,11038,7; 1339,4p=0,073(0 мес)*	1115,8941,1; 1409,6	1129,0896,0; 1284,5p=0,130(0 мес)*	1241,01137,9; 2009,0	1152,0899,0; 1294,0p=0,038(0 мес)*
sTNFα-R2, пг/млМедиана [ 25; 75]	3166,81874,2; 4181,8	2041,01816,8; 3496,9p=0,043,(0 мес)*	2221,31826,0; 2754,3	2265,31710,5; 2383,5p=0,875(0 мес)*	3040,02264,0; 3936,7	3246,02078,0; 3708,0p=0,255(0 мес)*
sIL-2R, пг/млМедиана [ 25; 75]	130,9102,8; 478,9	40,920,9; 55,0p=0,043 (0мес+)*	97,160,9; 247,0	90,8232,9; 247,0p=0,715 (0мес+)*	160,475,4; 437,3	49,538,0; 106,5p=0,067(0мес)*
sIL-6R, пг/млМедиана [ 25; 75]	166,0141,5; 192,5	145,7110,2; 163,7p=0,043, (0мес)*	170,7140,3; 208,7	182,5141,5; 215,2p=0,500 (0мес)*	193,6157,0; 206,5	163,7147,5; 173,01p=0,144 (0мес)*

Через 12 мес были обследованы пациенты с улучшением — 12 пациентов (50%), без динамики — 10 пациентов (41,7%). У пациентов с ухудшением течения ЭОП из-за малой выборки (2 пациента) анализ не проводился. Пациенты находились в неактивной фазе. Объем ЩЖ в обеих группах достоверно снизился. Уровень ТТГ нормализовался. Уровень антител к рТТГ оставался высоким. Уровень TGF-β1 в обеих группах достоверно снизился. Достоверных изменений уровней sTNFα-R1 и sTNFα-R2, sIL-2R и sIL-6R не произошло.

## ОБСУЖДЕНИЕ

Действие ионизирующего излучения связано с образованием свободных радикалов в среде микроокружения клеток. Свободные радикалы и оксиданты взаимодействуют с молекулами ДНК, вызывая большое количество разнообразных нарушений ее структуры, обеспечивая локальную деструкцию тиреоцитов [22]. Разрушающее действие 131I на ткань ЩЖ оказывают бета-частицы, которые обладают небольшой длиной пробега в тканях (1–3 мм) [1]. В литературе широко обсуждается вопрос о возможном ухудшении течения ЭОП после проведения РЙТ. Главным лимитирующим фактором проведения РЙТ БГ является активность аутоиммунного процесса в орбитах. Согласно клиническим рекомендациям EUGOGO, ETA, ATA [ 2021], лечение БГ в активную фазу ЭОП (CAS 5–7) только консервативное. В неактивную фазу (CAS 0–2) лечение БГ не отличается от лечения пациентов без ЭОП. В низко активную фазу (CAS 3–4) после РЙТ рекомендуется прием преднизолона с целью профилактики обострения ЭОП [20]. В нашем исследовании глюкокортикоиды (ГК) не назначались в связи с дизайном исследования.

ЭОП — это хроническое аутоиммунное заболевание орбиты, осложняющее течение БГ. Ключевую роль в иммунопатогенезе аутоиммунных заболеваний, в том числе и ЭОП, играют цитокинопосредованные механизмы [17]. Цитокины — важнейшие участники сигнальных путей клеток, обеспечивающие процессы регуляции клеточного роста, обмена веществ, выживаемости и апоптоза клеток, межклеточное и межсистемное взаимодействие, позитивную и негативную иммунорегуляцию [23]. Орбитальные фибробласты продуцируют TGF-β1, который стимулирует выработку гликозаминогликанов, а также дифференцировку орбитальных фибробластов в миофибробласты, что определяет развитие фиброза, особенно на поздних стадиях заболевания [24]. С другой стороны, TGF-β1 подавляет экспрессию рТТГ на фибробластах [25]. TGF-β1 оказывает преимущественно супрессорное влияние на динамику иммунного ответа за счет противовоспалительного эффекта, защищающего организм от избыточной продукции макрофагами и другими клетками воспаления цитотоксических соединений, и индуцирует образование Т регуляторных клеток (Тregs) на периферии. TGF-β1 участвует в регуляции иммунного ответа при аутоиммунной патологии ЩЖ, активируя адаптивные Тregs. В предыдущем исследовании цитокинового профиля у пациентов с ухудшением течения ЭОП или отсутствием эффекта на фоне проведения иммуносупрессивной терапии высокими дозами ГК мы выявили более низкие показатели цитокина TGF-β1 исходно и их снижение в процессе лечения, что позволило рассматривать данный цитокин как предиктор эффективности лечения ЭОП и прогноза заболевания [18]. В нашем исследовании у пациентов с ухудшением течения ЭОП через 3 и 6 мес после РЙТ было выявлено снижение показателя TGF-β1 от исходного по сравнению с пациентами с положительной динамикой ЭОП, а также достоверно более высокий уровень антител к рТТГ. В неактивную фазу ЭОП уровень TGF-β1 приближался к нормальному показателю [18], что свидетельствует о регрессии аутоиммунного процесса.

Роль солюбилизированных рецепторов цитокинов до конца не определена. Связывание растворимого рецептора с его лигандом может подавлять его биологическую активность и снижать провоспалительный эффект цитокина. С другой стороны, увеличение уровня растворимого рецептора противовоспалительного цитокина может явиться компенсаторной реакцией на его дефицит. В нашем исследовании растворимый рецептор провоспалительного цитокина sIL-6R снижался на уровне статистической тенденции у пациентов с ухудшением течения ЭОП после РЙТ. Уровень растворимого рецептора противовоспалительного цитокина sIL-2R достоверно снижался у пациентов с ухудшением и без динамики ЭОП после РЙТ, что может указывать на активацию аутоиммунного процесса в связи со снижением уровня IL-2. Уровень растворимого рецептора провоспалительного цитокина TNFа (sTNFа-R2), который экспрессируется в определенных популяциях лимфоцитов, включая T-регуляторные клетки, достоверно снижался у пациентов с ухудшением течения ЭОП через 3 и 6 мес после РЙТ. Уровень sTNFа-R1, который стабилизирует TNFα и увеличивает период его полураспада, запускает апоптоз, не имел однозначной тенденции и достоверно снижался у пациентов с ухудшением и улучшением течения ЭОП через 3 мес после РЙТ.

## ЗАКЛЮЧЕНИЕ

Цитокины играют важную роль в развитии, прогрессировании и регрессе ЭОП. На сегодняшний день имеются данные о роли цитокинов в развитии и поддержании воспаления при аутоиммунных заболеваниях, но они достаточно противоречивы и не позволяют однозначно объяснить их патологическое значение. На РЙТ направляются пациенты в неактивную или низкоактивную фазу. Лечение тиреотоксикоза в активную фазу ЭОП только консервативное. При невозможности приема тиреостатических препаратов (аллергические реакции, токсический гепатит, лейкопения, агранулицитоз) или при наличии компрессионного синдрома проводится тиреоидэктомия. Проведение РЙТ у пациентов с БГ и ЭОП в неактивной фазе или низкоактивной фазе снижает риск активации аутоиммунного воспаления в орбите. С другой стороны, минимальное изменение активности ЭОП после РЙТ всего на 1–2 балла по шкале CAS снижает результативность полученных данных. Наиболее показательной является динамика TGF-β1. Более низкие исходные показатели TGF-β1 и их достоверное снижение после РЙТ у пациентов с ухудшением течения ЭОП, по сравнению с пациентами с положительной динамикой ЭОП, позволяют использовать TGF-β1 в качестве биомаркера активации аутоиммунного процесса. Растворимые рецепторы sTNFα-R1 и sTNFα-R2, sIL2R и sIL-6R способны нейтрализовать цитокины, препятствуя их доступу к мембранным рецепторам и таким образом выполнять функции конкурирующих антагонистов цитокинов. Снижение или повышение их концентрации будет определять дисбаланс про- и противовоспалительных цитокинов в сыворотке крови и определять персистенцию активности ЭОП. Предикторами ухудшения течения ЭОП после РЙТ также являются персистенция высокого уровня антител к рТТГ и некомпенсированный гипотиреоз.

## ДОПОЛНИТЕЛЬНАЯ ИНФОРМАЦИЯ

Источник финансирования. Данная работа выполнена в соответствии с планом государственного задания. Регистрационный номер 123021000041-6.

Конфликт интересов. Авторы декларируют отсутствие явных и потенциальных конфликтов интересов, связанных с публикацией настоящей статьи.

Участие авторов. Все авторы внесли значимый вклад в проведение исследования и подготовку статьи, прочли и одобрили финальную версию статьи перед публикацией.
